# Epithelial-mesenchymal transition status of circulating tumor cells in breast cancer and its clinical relevance

**DOI:** 10.20892/j.issn.2095-3941.2019.0118

**Published:** 2020-02-15

**Authors:** Jiaojiao Zhou, Xuan Zhu, Shijie Wu, Jingxin Guo, Kun Zhang, Chunjing Xu, Huihui Chen, Yuxi Jin, Yuting Sun, Shu Zheng, Yiding Chen

**Affiliations:** ^1^Department of Surgical Oncology, the Second Affiliated Hospital, Zhejiang University School of Medicine, Hangzhou 310000, China; ^2^The Key Laboratory of Cancer Prevention and Intervention, China National Ministry of Education, Zhejiang University School of Medicine, Hangzhou 310000, China; ^3^Life Science Institute, Zhejiang University, Hangzhou 310000, China

**Keywords:** Circulating tumor cells, breast cancer, epithelial-to-mesenchymal transition, estrogen receptor/human epidermal growth factor receptor 2 expression, support vector machine algorithm

## Abstract

**Objective:** Circulating tumor cells (CTCs) play a critical role in cancer metastasis, but their prevalence and significance remain unclear. This study attempted to track the epithelial-mesenchymal transition (EMT) status of CTCs in breast cancer patients and investigate their clinical relevance.

**Methods:** In this study, the established negFACS-IF:E/M platform was applied to isolate rare CTCs and characterize their EMT status in breast cancer. A total of 89 breast cancer patients were recruited, including stage 0–III (*n* = 60) and late stage (*n* = 29) cases.

**Results:** Using the negFACS-IF:E/M platform, it was found that in human epidermal growth factor receptor 2 (HER2)+ patients, mesenchymal CTCs usually exhibited a high percentage of HER2+ cells. Stage IV breast cancer patients had considerably more CTCs than stage 0–III patients. Among stage 0–III breast cancers, the HER2 subtype included a significantly higher percentage of mesenchymal and biphenotypic (epithelial and mesenchymal) CTCs than the luminal A or B subtypes. Among stage IV patients, CTCs were predominantly epithelial in cases with local recurrence and were more mesenchymal in cases with distant metastasis. By applying a support vector machine (SVM) algorithm, the EMT status of CTCs could distinguish between breast cancer cases with metastasis/local recurrence and those without recurrence.

**Conclusions:** The negFACS-IF:E/M platform provides a flexible and generally acceptable method for the highly sensitive and specific detection of CTCs and their EMT traits in breast cancer. This study demonstrated that the EMT status of CTCs had high clinical relevance in breast cancer, especially in predicting the distant metastasis or local recurrence of breast cancer.

## Introduction

Breast cancer is the leading cause of cancer-related deaths in women^1^. Most breast cancer patients die of distant metastasis, and the median survival of patients with metastatic breast cancer is 18–26 months^2^. Hematogenous dissemination of circulating tumor cells (CTCs) from the primary tumor is a crucial step in the metastasis cascade. The monitoring of CTCs has recently emerged as a promising approach for the detection of early disease, assessment of prognosis, and evaluation of therapeutic response in breast cancer^3^.

The multi-center research by Cristofanilli et al.^4,5^ found that the number of CTCs was highly predictive of cancer progression and survival in metastatic breast cancer. Subsequently, a large number of studies further validated the value of CTCs for prognostic prediction in metastatic and primary breast cancer^6–8^. Some research depicted that CTC assessment was equivalent to or even more sensitive than current imaging methods^9^. CTCs were proven to be influenced by the treatment, thus monitoring the response of CTCs allows the evaluation of treatment efficacy and detection of disease progression in breast cancer patients^10,11^.

The enrichment and isolation of CTCs is difficult due to their very low concentration in the peripheral blood^12^. In addition to the cells’ rarity, the heterogeneity of CTCs renders their detection challenging. The classic test for CTCs (CellSearch®) was intended to enumerate CTCs of epithelial origin^13^. However, considering the large number of breast CTCs with mesenchymal phenotypes, this approach may not be suitable for breast cancer^14,15^. Various microfluidic devices for capturing CTCs have been developed^16–18^. Nevertheless, most of these devices remain confined to individual laboratory usage, owing to the complexity and high cost associated with their manufacture.

To obtain a comprehensive understanding of the dynamic molecular features of CTCs in breast cancer, we developed a strategy (negFACS-IF:E/M) that combines the strength of isolating rare CTCs with the benefits of epithelial-to-mesenchymal transition (EMT) characterization in breast cancer. The procedures involved in the negFACS-IF:E/M strategy are simple and do not require a microfluidic device, rendering this method feasible for general application. Importantly, this strategy is also cost-effective (i.e., $70–$80 per test) *vs.* other currently available commercial CTC testing. In the present study, this strategy was used to further demonstrate the heterogeneity of breast CTCs, such as the estrogen receptor (ER) and human epidermal growth factor receptor 2 (HER2) subtypes. In addition, the clinical significance of the detected epithelial CTCs (E CTCs) and mesenchymal CTCs (M CTCs) is discussed.

## Materials and methods

### Patient samples and blood collection

Peripheral blood samples were collected from breast cancer patients with or without metastasis. Five blood samples from healthy donors were also included in this study. Written informed consent was provided by all patients prior to the collection of blood samples. This study was approved by the Medical Ethics Committees of the Second Affiliated Hospital of Zhejiang University School of Medicine. A total of 89 breast cancer patients were recruited to analyze the EMT status of their CTCs, including stage 0–III (*n* = 60) and late stage (*n* = 29) cases. Blood samples from stage 0–III patients were collected before any treatment (including surgery, chemotherapy, radiology, endocrine therapy, and target therapy, etc.). The sampling timing of late stage patients was after the patients were diagnosed to have local recurrence or distant metastasis. Five healthy donors and five patients with fibroadenoma were also included as healthy and benign controls. The inclusion criteria were: female breast cancer patients with invasive ductal carcinoma (IDC) and the stage 0 disease referred to ductal carcinoma *in situ* (DCIS). The exclusion criteria were: male breast cancer, inflammatory breast cancer, patients with a blood disease, a vascular disease, immunological disorders, or a history of other cancer. Blood samples were collected in vacuum blood collection tubes with anticoagulant ethylenediaminetetraacetic acid (EDTA). All blood samples were stored at 4 °C and processed within 3 h of withdrawal. Clinical characterizations of patients used in the study are listed in **Supplementary Tables S1** and **S2**.

### Cell culture

T-47D was obtained from the American Type Culture Collection (ATCC, Manassas, VA, USA), while the other cell lines were obtained from the Cell Bank of Type Culture Collection of the Chinese Academy of Science, Beijing, China, which was also imported by the Institute of Cell Biology from the ATCC. All cell lines passed the cell line authentication assessment. The culture conditions of the breast cancer cell lines MCF-7, T-47D, BT-474, SK-BR-3, MDA-MB-231, MDA-MB-468, Hs 578T, and BT-549 followed the protocol provided by the ATCC.

### CTC enrichment

Initially, CTCs were enriched through density gradient centrifugation coupled with flow cytometry. Briefly, 4 mL of whole blood was diluted with 4 mL of phosphate-buffered saline (PBS) and gently layered into a centrifuge tube (Corning Inc, Corning, USA) containing 4 mL Ficoll-Paque Premium sterile solution (GE-Healthcare, Uppsala, Sweden). After density gradient centrifugation (1,040 × *g*, 20 °C, 15 min) without breaking, the interphase consisting of peripheral blood mononuclear cells and CTCs was transferred to a clean tube and washed using PBS. Red blood cell lysis was performed using ammonium-chloride-potassium (ACK) lysing buffer (Boster Biological Technology, Ltd, Wuhan, China). Subsequently, the enrichment was performed by incubating with CD45-PE-Cy5 (#555484; Becton Dickinson Pharmingen, San Diego, CA, USA) and isotype control (#555750; Becton Dickinson Pharmingen) antibody, and CTCs were negatively enriched *via* hematopoietic cell (CD45^+^) depletion through flow cytometry (BD FACSAria^™^ IIu; Becton Dickinson). During flow cytometry, the main cell population was gated based on their forward and side scatter properties to avoid the interference of the cell debris. In the gating strategy, isotype control for each blood sample was used to gate the PE-Cy5 negative area.

### Identification of CTC subtypes through immunofluorescence staining

After enrichment, immunofluorescence staining of epithelial and mesenchymal markers was performed in the CD45^+^-depleted pre-enriched CTCs. The enriched cells were fixed in 4% paraformaldehyde for 15 min, washed with PBS, permeabilized with 0.1% Triton X-100 for 10 min, blocked with blocking solution containing 1% bovine serum albumin (BSA) for 1 h, bound with primary antibodies to epithelial and mesenchymal markers, overnight at 4 °C, washed in PBS, bound with secondary antibodies for 30 min at 37 °C, washed with PBS, stained with 4' 6-diamidino-2-phenylindole dihydrochloride (Sigma-Aldrich, St. Louis, MO, USA) for 10 min, and stored in the dark at 4 °C until evaluation. All of the primary and secondary antibodies used are listed in **Supplementary Table S3**.

### Immunocytochemistry staining of CTCs

Immunocytochemistry staining was performed using the avidin-biotin-peroxidase complex method according to the manufacturer’s protocol (UltraSensitive S-P; Maxim Biomedical, China). The cells on the slides were incubated with primary antibodies for ER (#790-4325; Ventana, Oro Valley, AZ, USA) or HER2 (#790-4493; Ventana) at room temperature for 32 min, followed by incubation with a peroxidase polymer labeled secondary antibody. Subsequently, peroxidase activity was determined using diaminobenzidine. Finally, slides with cells were counterstained using hematoxylin.

### Single cell micromanipulation and quantitative reverse transcription polymerase chain reaction (qRT-PCR)

After micromanipulation, the generation of full-length cDNA from a single cell was performed using a Discover-sc WTA Kit (#N711; Vazyme, Nanjing, China) according to the manufacturer’s protocol. The quantity and quality of the nucleic acid were measured using Qubit 4 Fluorometer® (Invitrogen, Carlsbad, CA, USA) and an Agilent 2100 Bioanalyzer® (Agilent, Santa Clara, CA, USA). The qRT-PCR program was as follows: 95 °C for 30 s and 40 cycles at 95 °C for 15 s, and 60 °C for 60 s using iTaq^™^ Universal SYBR Green Supermix (#172-5124, Bio-Rad, Hercules, CA, USA). The primers used are listed in **Supplementary Table S4**.

### Support vector machine (SVM) classifier

The SVM algorithm was performed using the e1071 R-package. stage 0–III first episode patients in the study were classified as nonmetastatic/local recurrence patients, whereas late-stage patients were classified as metastasis/local recurrence patients. Prior to the construction of the SVM algorithm, patients with low CTC numbers (i.e., total number of CTCs < 5) were excluded to reduce the noise and increase the robustness of the SVM model. In principle, the SVM algorithm determines the location of all samples in a high dimensional space, of which the three axes represent the numbers of E, M, and biphenotypic CTCs (E + M CTCs). Through the training cohort, the SVM algorithm drew a hyperplane that best separated the two classes. With this established hyperplane, patients with different numbers of E, M, and E + M CTCs could be positioned at each side of the hyperplane, classifying the patients into those with metastasis/local recurrence and those without. Patients in the validation cohort were randomly extracted from the training cohort. A receiver operating curve (ROC) was used to assess the predictive strength of the SVM algorithm, which was implemented using the R-package pROC (version 1.7.3).

### Statistical analysis

Statistical analysis was performed using SPSS 20.0 software (SPSS Inc., Chicago, IL, USA). The statistical significance of non-normally distributed data was analyzed using the Mann-Whitney U or Kruskal-Wallis tests. All statistical analyses were two-sided. *P* < 0.05 denoted statistical significance.

## Results

### Isolation and characterization of CTCs in breast cancer

**Figure 1:**
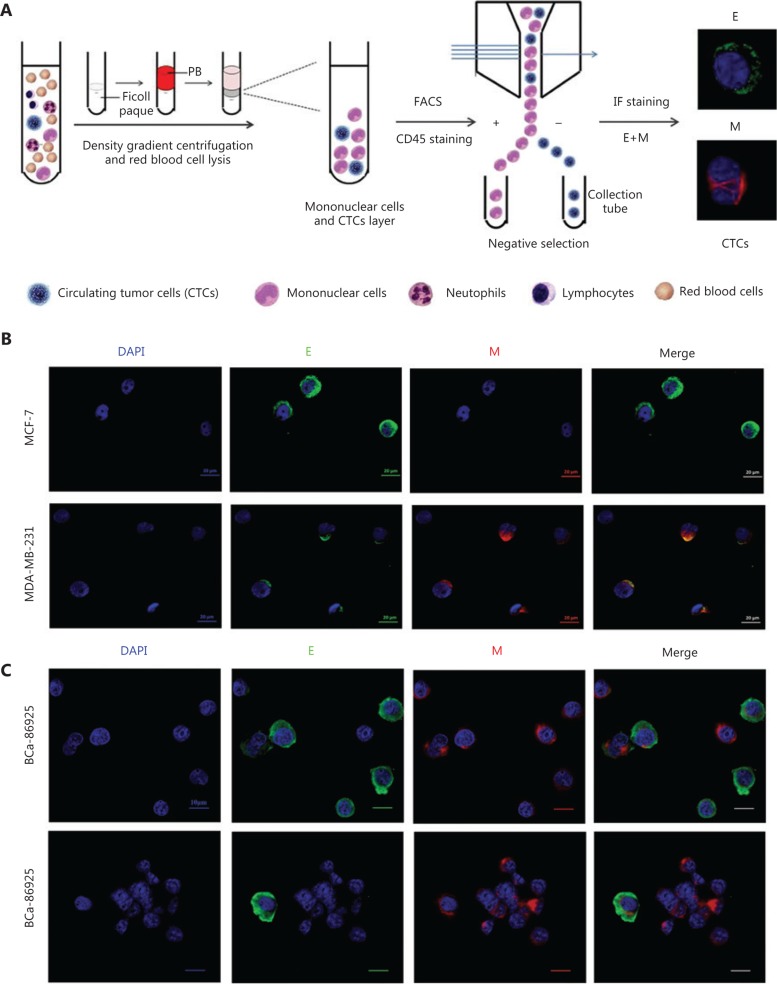
Schematic of the negFACS-IF: E/M platform and epithelial-mesenchymal transition (EMT) status of circulating tumor cells (CTCs) characterized by the negFACS-IF:E/M platform. (A) This negFACS-IF:E/M platform involves three main procedures: density gradient centrifugation to enrich the interphase layer consisting of peripheral blood mononuclear cells and CTCs; FACS for negative selection by depleting the peripheral blood cells (CD45+); and immunofluorescence staining to identify the rare CTCs and characterize their EMT status in breast cancer. (B) Performance of the negFACS-IF:E/M platform was initially validated in representative “epithelial (E)” MCF-7 and “mesenchymal (M)” MDA-MB-231 spiked into whole blood. (C) Representative images of CTCs in a breast cancer patient, defined using the negFACS-IF:E/M platform. The E CTCs prominently expressed epithelial markers but not mesenchymal markers. The M CTCs prominently expressed mesenchymal markers but not epithelial markers. The E + M CTCs expressed both epithelial and mesenchymal markers.

EMT in tumor cells, especially in CTCs, has been suggested as a critical component of the metastatic process in breast cancer. The negFACS-IF:E/M strategy that we developed in this study (**Figure 1A**) can identify and isolate the rare CTCs of dynamic EMT characterization in breast cancer. After density gradient centrifugation and negative selection *via* fluorescence-activated cell sorting (FACS), dynamic EMT characterization of the enriched CTCs was performed through immunofluorescence staining of epithelial and mesenchymal markers (**Figure 1A**).

The specificity of the antibodies for epithelial (EpCAM, E-cadherin, CK8, CK18, and CK19) and mesenchymal (vimentin, fibronectin, and N-cadherin) markers used in the negFACS-IF:E/M platform were initially validated in various breast cancer cell lines (**Supplementary Figure S1**). MDA-MB-231 and Hs 578T displayed high expression of “mesenchymal” markers, while less invasive cell lines, such as MCF-7, T-47D, and SK-BR-3, demonstrated the opposite expression profile.

The performance of the negFACS-IF:E/M platform was initially validated using the representative “epithelial (E)” MCF-7 and “mesenchymal (M)” MDA-MB-231 cell lines spiked into whole blood. The results showed that negFACS-IF:E/M platform can isolate both “epithelial” MCF-7 and “mesenchymal” MDA-MB-231 (**Figure 1B**). The EMT status of MCF-7 and MDA-MB-231 cells sorted using the negFACS-IF:E/M platform was consistent with that of the original cell lines. Performance recovery of MCF-7 and MDA-MB-231 cells through negFACS-IF:E/M platform was > 90% (mean value) (**Supplementary Figure S2**).

Subsequently, the negFACS-IF:E/M platform was applied to whole blood samples (4 mL) obtained from breast cancer patients. Both “epithelial” and “mesenchymal” CTCs from breast cancer patients could be detected (**Figure 1C**). According to the expression levels of epithelial and mesenchymal markers, breast cancer CTCs could be further divided into the following three subtypes (**Figure 1C**): E CTCs prominently expressing epithelial markers but not mesenchymal markers, M CTCs prominently expressing mesenchymal markers but not epithelial markers, and E + M CTCs expressing both epithelial and mesenchymal markers.

**Figure 2:**
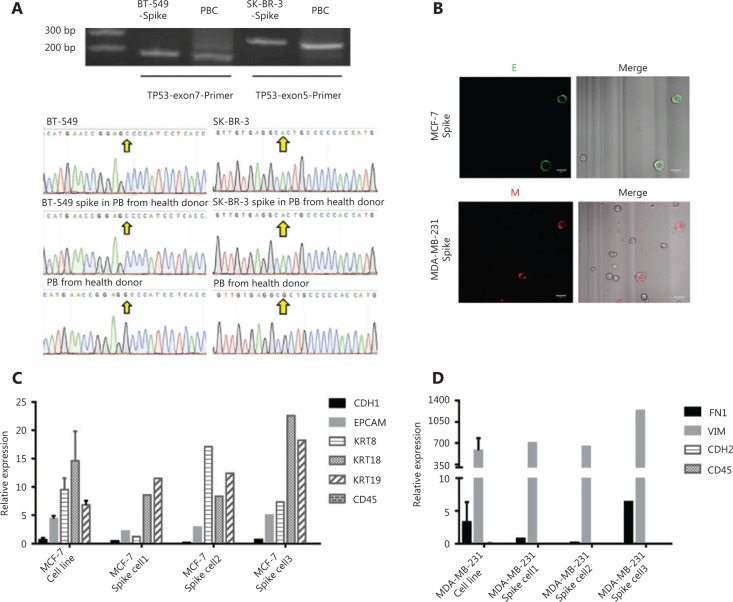
Validation of the specificity of circulating tumor cell (CTC) isolation using the negFACS-IF:E/M strategy at the DNA and single cell RNA expression levels. (A) Using the negFACS-IF:E/M strategy, TP53 mutations in spiked BT-549 and SK-BR-3 cells were accurately detected without the presence of nonspecific signals. (B) Representative images of live MCF-7 and MDA-MB-231 cells sorted for micromanipulation. (C) Single cell RNA expression of isolated MCF-7, profiled with a panel of “epithelial” -related transcripts (CDH1, EpCAM, KRT8, KRT18, and KRT19). (D) Isolated MDA-MB-231, profiled with a panel of “mesenchymal”-related transcripts (FN1, VIM, and CDH2), both of which were consistent with the MCF-7 and MDA-MB-231 original cell lines, as well as their “E” or “M” phenotype through immunostaining. CD45 expression was not detected in isolated MCF-7 and MDA-MB-231 cells.

To further confirm the accuracy of CTC isolation using the negFACS-IF:E/M strategy, we examined the capture specificity at the DNA level. As shown in the ATCC® database, tumor protein 53 mutations (TP53) in BT-549 (c.747G > C, at exon 7) and SK-BR-3 (c.524G > A, at exon 5) breast cancer cell lines were validated in our lab (**Figure 2A**). Using the negFACS-IF:E/M strategy, TP53 mutations in spiked BT-549 and SK-BR-3 breast cancer cells from the peripheral blood of healthy donors were accurately detected without nonspecific signals (**Figure 2A**).

Moreover, the specificity of the EMT characterization through the negFACS-IF:E/M strategy was validated at the single-cell RNA expression level. After density gradient centrifugation and FACS, the cells were stained with a mixture of conjugated anti-epithelial or a mixture of anti-mesenchymal antibodies (**Figure 2B**) to identify their “E” or “M” phenotype with the goal of obtaining live single cells for single-cell RNA expression analysis. Single MCF-7 (with “E” phenotype) or MDA-MB-231 (with “M” phenotype) CTCs were individually isolated through micromanipulation. Subsequently, the cells were subjected to RNA expression analyses *via* high-throughput single cell qPCR, profiling for a panel of transcripts implicated in epithelial (CDH1, EPCAM, KRT8, KRT18, and KRT19) or mesenchymal (FN1, VIM, and CDH2) cell fates. The results showed that the RNA expression of the sorted single MCF-7 or MDA-MB-231 CTCs was consistent with an “E” or “M” immunostaining (**Figure 2C and 2D**), demonstrating the specificity of the EMT characterization via the negFACS-IF:E/M strategy. Furthermore, all sorted MCF-7 or MDA-MB-231 CTCs did not express CD45, which allowed mononuclear cell contamination to be avoided.

Collectively, these findings demonstrated the flexibility and specificity of the negFACS-IF:E/M strategy to isolate rare CTCs in breast cancer patients and to identify their EMT characterization.

### ER and HER2 expression patterns in CTCs of breast cancer

**Figure 3:**
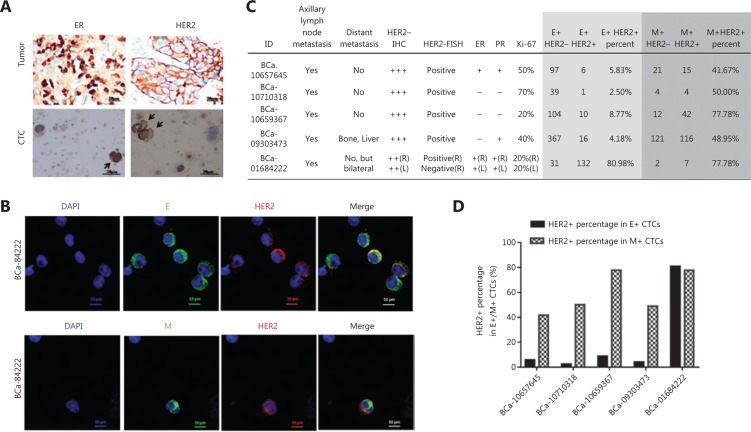
ER and HER2 expression patterns in circulating tumor cells (CTCs) of breast cancer. (A) Cytopathology analysis of CTCs (lower panel) matched to primary tumors (upper panel) in breast cancer patients through immunocytochemistry staining. Arrows point to an ER + CTC (left panel) and 2 HER2 + CTCs (right panel). (B) Representative images of the HER2 expression status in epithelial and mesenchymal CTCs, respectively, through immunofluorescence staining. (C, D) HER2 expression status in epithelial and mesenchymal CTCs in five first episode HER2+ patients who had not undergone clinical treatments.

We further confirmed the ER and HER2 status in CTCs. Initially, we stained the enriched CTCs through immunocytochemistry staining used for cytopathology analysis in clinical laboratories. Compared with the primary tumor, the enriched CTCs presented a similar morphological appearance (**Figure 3A**). The ER+ and HER2+ CTCs were also evident in patients with luminal and HER2 breast cancer, respectively (**Figure 3A**).

The expression of HER2 in E CTCs and M CTCs was further investigated through immunofluorescence staining (**Figure 3B**). Five first episode HER2+ patients who had not undergone any clinical treatment were recruited. Intriguingly, all HER2+ patients exhibited a high percentage of HER2+ cells in their M CTCs [mean ± standard deviation (SD), 59.24% ± 17.23%]. In four of the selected HER2+ patients (BCa-10657645, BCa-10710318, BCa-10659367, and BCa-09303473), M CTCs had a clearly higher percentage of HER2 expression compared to that in E CTCs (**Figure 3C and 3D**), indicating that HER2 was likely to be expressed in mesenchymal (M) CTCs. In the case of synchronous bilateral breast cancer (BCa-01684222), HER2 expression was high in both E CTCs and M CTCs (**Figure 3C and 3D**). It is difficult to determine whether the high HER2 expression in E CTCs was related to the left breast tumor, which was of the luminal subtype (i.e., ER + PR + HER2–). Collectively, these results confirmed the expression of HER2 in CTCs and revealed that in HER2+ patients, M CTCs usually exhibit a high percentage of HER2+ cells.

### Correlations between CTC features and clinical characteristics of breast cancer

**Figure 4:**
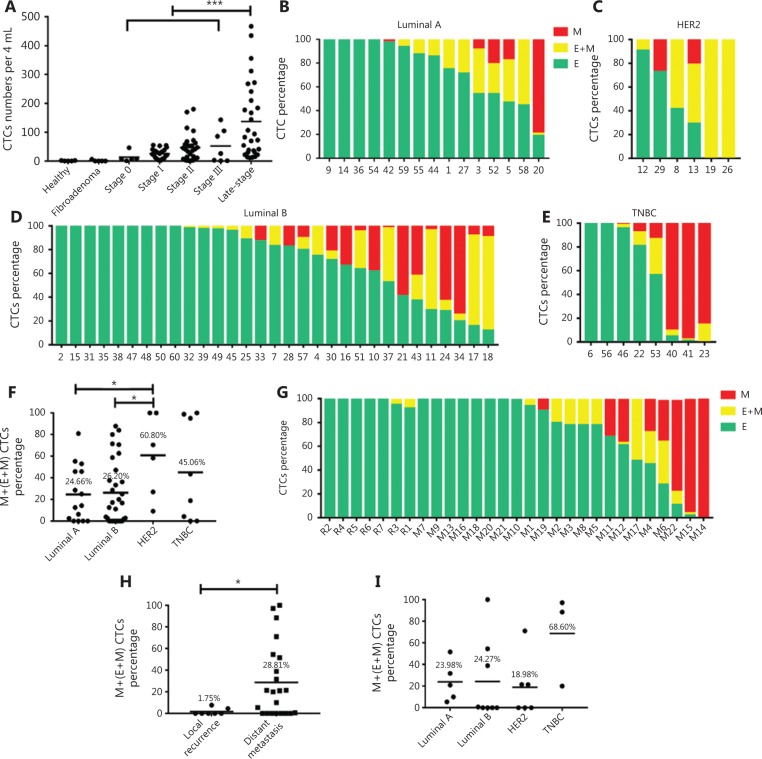
Correlations between circulating tumor cell (CTC) features and clinical characteristics of breast cancer. (A) Numbers of CTCs among healthy donors, patients with fibroadenoma, and breast cancer patients at different stages. The number of CTCs was the sum of the E, M, and E + M CTC subtypes. (B–E) Percentage of E, E + M, and M CTCs in luminal A, luminal B, HER2, and triple negative breast cancer. (F) Quantitation of epithelial-mesenchymal transition (EMT) features in CTCs among different subtypes of breast cancer. (G) Percentage of E, E + M, and M CTCs in late stage breast cancers, with local recurrence (No. R1-R7) or distant metastasis (No. M1-M22). (H) Quantitation of EMT features in CTCs between patients with local recurrence and distant metastasis. (I) Quantitation of EMT features in CTCs among different subtypes of patients with distant metastasis. *P < 0.05; ***P < 0.001.

Next, we investigated the prevalence of CTCs in breast cancer patients at different stages. In this study, the number of CTCs was the sum of E, M, and E + M CTC subtypes. As shown in **Figure 4A**, CTCs were rare in healthy women (mean, 0.8 per 4 mL of whole blood) and in patients with fibroadenoma (i.e., benign breast disease) (mean, 1.2 per 4 mL of whole blood). None of the normal control donors exhibited more than seven CTC-like cells per 4 mL of blood. At the time of enrollment in the study, at least seven CTCs per 4 mL of blood were detected in 86.52% of the breast cancer patients. Moreover, all patients with late stage breast cancer exhibited more than seven CTCs per 4 mL of blood. The CTC numbers of stage 0–III breast cancer represented the baseline level prior to any treatment. It was obvious that late stage breast cancer had notably more CTCs than stage 0–III disease (mean number per 4 mL of whole blood, 137.48 *vs.* 37.42, respectively, *P* < 0.001) (**Figure 4A**). Moreover, in stage 0–III breast cancer, there was an increasing trend in the number of CTCs in parallel with the increasing stage of cancer (*P* > 0.05) (**Figure 4A**). Therefore, a larger number of CTCs may indicate the presence of more aggressive tumors in breast cancer.

Subsequently, we assessed the EMT status of CTCs among different subtypes of breast cancer (**Figure 4B–E**) by determining the (M and E + M)/total CTC ratios. The (M and E + M)/total CTC ratios were quantitated by calculating the percentage of M and E + M CTCs. Compared with luminal breast cancer, the HER2 subgroup exhibited a significantly higher percentage of M and E + M CTCs per 4 mL of blood (mean, HER2: 60.80% *vs.* luminal A: 24.66%, *P* < 0.05; HER2: 60.80% *vs.* luminal B: 26.20%, *P* < 0.05) (**Figure 4F**). These results indicated the high malignancy of HER2 breast cancer.

Interestingly, among the late-stage breast cancer patients, the (M and E + M)/total CTC ratios of cases with distant metastasis were significantly higher than those of cases with local recurrence (mean, 28.81% *vs.* 1.75%, respectively, *P* < 0.05) (**Figure 4G and H**). This finding revealed that the CTCs obtained from late stage patients with local recurrence were predominantly epithelial, whereas those obtained from patients with distant metastasis were mesenchymal. However, the (M and E + M)/total CTC ratios were not significantly different among the different subtypes in late stage patients with distant metastasis (*P* > 0.05) (**Figure 4I**).

### The EMT status of CTCs enables the prediction of metastasis or local recurrence

Next, to evaluate the predictive capacity of the EMT status of CTCs for metastasis or local recurrence in breast cancer, we developed a classification using the training cohort (*n* = 77). This classification was achieved by employing a leave-one-out cross-validation SVM algorithm, in which the three variables represented the numbers of E CTCs, M CTCs, and E + M CTCs, respectively. SVM is an algorithm previously used to classify primary and metastatic tumor tissues^19–21^. Briefly, the unsupervised SVM algorithm classifies each individual sample as metastasis/local recurrence or not *via* comparison with all other samples (77-1) and was performed 77 times to classify and cross-validate all individual samples.

**Figure 5:**
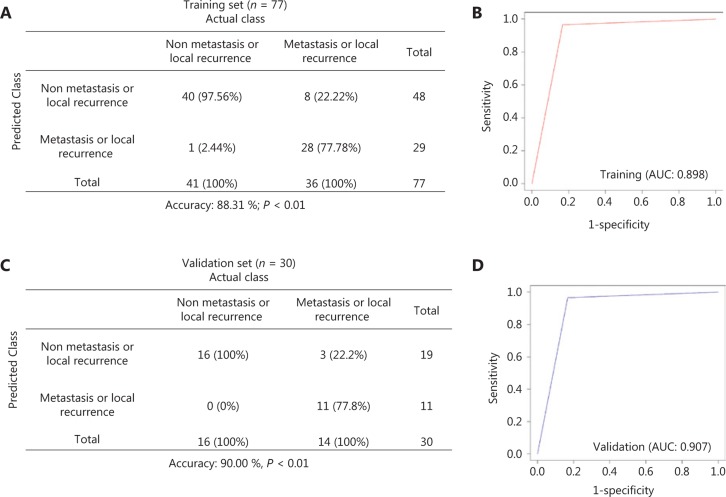
Support vector machine (SVM) diagnostics of metastasis or local recurrence based on the epithelial-mesenchymal transition (EMT) status of circulating tumor cells (CTCs). (A) Cross-table of SVM diagnostics of metastasis/local recurrence in the training cohort (*n* = 77). Data include the sample numbers and detection rates in percentages. (B) The receiver operating curve (ROC) of SVM diagnostics in the training cohort. (C) Performance of the SVM algorithm in the validation cohort (*n* = 30). The data include the sample numbers and detection rates in percentages. (D) The ROC curve of SVM diagnostics in the validation cohort.

To reduce the noise and increase the robustness of the SVM model, 77 patients with more than five CTCs were included in the model. For the training cohort (*n* = 77), the model yielded a sensitivity of 77.78%, a specificity of 97.56%, and an accuracy of 88.31% (**Figure 5A**) with an area under the curve (AUC) of 0.898 to detect metastasis or local recurrence in breast cancer (**Figure 5B**). Subsequent validation obtained using a sampling validation cohort (*n* = 30) randomly extracted from the training cohort yielded a sensitivity of 77.80%, a specificity of 100%, and an accuracy of 90.00% (**Figure 5C**) with an AUC of 0.907 (**Figure 5D**). The training and validation sets yielded similar accuracy (mean overall accuracy: 89.16% ± 1.20%), confirming reproducible classification accuracy in the dataset. This finding suggested the predictive potential of the EMT status of CTCs, as determined by the SVM algorithm, in distinguishing between breast cancer cases with metastasis/local recurrence and those without.

## Discussion

Classic CTC assays positively select CTCs by targeting epithelial markers (e.g., EpCAM)^13^. However, CTCs of breast cancer are heterogeneous, and EMT has been invoked as a root cause of this heterogeneity^22,23^. Our negFACS-IF:E/M platform provided evidence of EMT in CTCs of breast cancer patients. The negFACS-IF:E/M platform is applicable for general use, as the main procedures of this platform can be performed in most laboratories. In addition, this platform is flexible and convenient for the analysis of other important characteristics of breast cancer CTCs, such as ER/HER2 expression, DNA mutations and RNA expression profiles.

Differences in ER and HER2 status between the primary lesion and the subsequent recurrent/metastatic site have been frequently reported at a rate of 10% to 30%^24^. The expression of ER and HER2 in CTCs is particularly important in cases in which a repeat tumor biopsy in the recurrent/metastatic site is unavailable. Our negFACS-IF:E/M platform was able to characterize the ER and HER2 expression pattern in breast CTCs. We observed that HER2 was highly expressed in mesenchymal CTCs in HER2+ breast cancer patients. A previous study using the CellSearch® assay to isolate CTCs showed that primary tumors and CTCs displayed a concordant HER2 status in 59% of breast cancer cases, and this value in metastases and CTCs was 67%^25^. In this context, the use of the negFACS-IF:E/M strategy may improve the discrepancy of HER2 status between CTCs and primary tumor/metastases by evaluating the HER2 status in mesenchymal CTCs.

The CellSearch® system had a low CTC detection rate, with CTC positivity in metastatic disease ranging between 20% and 50%^26^. It has been reported that the expression of EpCAM in CTCs can be downregulated by 10-fold compared with that observed in primary or metastatic sites^27^. This finding implies the insufficient efficiency of epithelial marker-based positive selection. The EMT status is usually depicted by a panel of canonical epithelial and mesenchymal markers. However, the expression of different epithelial and mesenchymal markers can vary considerably in breast cancer cells (**Supplementary Figures S3 and S4**). Therefore, we used multiple markers for detection, which defined the epithelial phenotype of CTCs as expressing either cytokeratins, EpCAM, or E-cadherin, while defining the mesenchymal phenotype as expressing either vimentin, fibronectin, or N-cadherin. In our study, the CTC count was the sum of the E CTCs, M CTCs, and E + M CTCs subtypes. Hence, the reported CTC positivity in this study was higher than that observed in other studies, such as those using the CellSearch® system^8,28^. Nevertheless, CTCs in healthy donors and patients with benign fibroadenoma remained very rare. Consistent with previous studies^8,29^, the CTC count in this study was associated with clinical parameters, including tumor stage and the presence of distant metastasis. Notably, there is a debate regarding the role of “EMT-like CTCs” as circulating tumor-derived cells (CTDCs, due to the overlapping expression of mesenchymal markers between real EMT CTCs and CTDCs (i.e., circulating macrophages, fibroblasts, and endothelial cells)^30^. However, the evidence for the existence and biological significance of these CTDCs is currently very limited.

Analyses of the EMT status in CTCs of different breast cancer subtypes showed that CTCs from treatment-naïve patients with the HER2 subtype were mostly mesenchymal. This finding supports previous findings indicating that HER2 amplification is associated with an increased risk of metastasis and poor outcome^31,32^. Compared with CTCs “frozen” in the epithelial or mesenchymal phenotype, CTCs with an “intermediate phenotype” may be more important in metastasis due to their higher degree of plasticity and stemness^16,33^. Consistent with this finding, in our study, the aggressive HER2 subtype exhibited the highest percentage of E + M CTCs, which can be regarded as the “intermediate phenotype”. However, the (M and E + M)/total CTC ratios were not significantly different among the different subtypes in late-stage patients with distant metastasis. Thus, more patients are needed to be recruited for the further analysis on CTC features in late-stage patients with distant metastasis. Moreover, we noted that CTCs of local recurrent disease demonstrated a preferential expression of epithelial markers, whereas those metastatic stage IV diseases preferentially expressed mesenchymal markers. This phenomenon may partially reflect the dominant properties of proliferation in local recurrent tumor cells and the significant features of invasion in distant metastatic tumor cells. Consequently, the EMT status of CTCs may provide clues regarding the patterns of recurrence (i.e., local recurrence or distant metastasis).

By applying the SVM algorithm in this study, the number of E, E + M, and M CTCs could accurately predict the distant metastasis or local recurrence of breast cancer. The AUC-ROC values of the training and validation cohorts were 0.898 and 0.907, respectively, displaying good predictive power. To the best of our knowledge, this study is the first to use this approach to assess the predictive power of CTC subpopulations in breast cancer patients with distant metastasis or local recurrence.

## Conclusions

In summary, our data suggested that the negFACS-IF:E/M platform had the ability to isolate and characterize the EMT status of breast cancer CTCs with high efficiency and feasibility. This platform was also able to analyze other important features of CTCs, broadening its potential application in clinical and basic research. Through this platform, the EMT status of breast cancer CTCs was found to be closely associated with cancer subtypes, form of recurrence, and metastatic status. By applying the SVM algorithm, the number of E, E + M, and M CTCs predicted the development of distant metastasis or local recurrence of breast cancer. Nevertheless, our study had several limitations. First, this negFACS-IF:E/M platform required a skilled/trained operator to perform the three main procedures carefully and smoothly. Second, the use of a cocktail of antibodies, including CD45, may result in a greater depletion of leukocytes of different lineages. Third, more patients with longitudinal monitoring of CTCs are needed to further validate the clinical utility of this platform. We envision that the technology for the detection of CTCs will continue to improve in terms of specificity and sensitivity. This approach may be used in the early detection of invasive cancer before the development of metastases.
