# Supplementary material for: Epithelial-mesenchymal transition status of circulating tumor cells in breast cancer and its clinical relevance
Source: Cancer Biol Med. 2020 Feb 15;17(1):169–80. doi: 10.20892/j.issn.2095-3941.2019.0118 (PMC7142848; doi:10.20892/j.issn.2095-3941.2019.0118)
Supplement: Supplementary file 1 [file cbm-17-169-s001.pdf]

Supplementary materials

Table S1 Clinical characterization of the stage 0–III patients included in the study

Patient ID	Histology	Stage	TNM	Subtype	Data used in figures
1	IDC	I	T1N0M0	Luminal A	Figures 4 and 5
2	IDC	I	T1N0M0	Luminal B	Figure 4
3	IDC	I	T1N0M0	Luminal A	Figures 4 and 5
4	IDC	II	T2N1M0	Luminal B	Figure 4
5	IDC	I	T1N0M0	Luminal A	Figures 4 and 5
6	IDC	II	T2N0M0	TNBC	Figures 4 and 5
7	IDC	I	T1N0M0	Luminal B	Figures 4 and 5
8	IDC	I	T1N0M0	HER2	Figures 4 and 5
9	IDC	II	T2N0M0	Luminal A	Figure 4
10	IDC	I	T1N0M0	Luminal B	Figures 4 and 5
11	IDC	II	T2N0M0	Luminal B	Figures 4 and 5
12	IDC	II	T1N1M0	HER2	Figures 4 and 5
13	IDC	II	T2N1M0	HER2	Figures 4 and 5
14	DCIS	0	TisN0M0	Luminal A	Figure 4
15	DCIS	0	TisN0M0	Luminal B	Figures 4 and 5
16	IDC	III	T1N3M0	Luminal B	Figure 4
17	IDC	II	T2N1M0	Luminal B	Figures 4 and 5
18	IDC	II	T2N0M0	Luminal B	Figures 4 and 5
19	IDC	III	T1N2M0	HER2	Figure 4
20	IDC	III	T2N2M0	Luminal A	Figures 4 and 5
21	IDC	II	T2N1M0	Luminal B	Figures 4 and 5
22	IDC	I	T1N0M0	TNBC	Figures 4 and 5
23	IDC	I	T1N0M0	TNBC	Figures 4 and 5
24	IDC	I	T1N0M0	Luminal B	Figures 4 and 5
25	IDC	I	T1N0M0	Luminal B	Figures 4 and 5
26	IDC	III	T4N1M0	HER2	Figure 4
27	IDC	II	T1N1M0	Luminal A	Figures 4 and 5
28	IDC	II	T2N0M0	Luminal B	Figures 4 and 5
29	IDC	III	T3N1M0	HER2	Figures 4 and 5
30	IDC	II	T2N0M0	Luminal B	Figures 4 and 5
31	IDC	II	T2N0M0	Luminal B	Figure 4
32	IDC	II	T1N1M0	Luminal B	Figures 4 and 5
33	IDC	I	T1N0M0	Luminal B	Figures 4 and 5
34	IDC	I	T1N0M0	Luminal B	Figures 4 and 5

Table S1 Continued

Patient ID	Histology	Stage	TNM	Subtype	Data used in figures
35	IDC	II	T2N0M0	Luminal B	Figures 4 and 5
36	IDC	I	T1N0M0	Luminal A	Figure 4
37	IDC	II	T2N1M0	Luminal B	Figures 4 and 5
38	IDC	II	T2N0M0	Luminal B	Figure 4
39	IDC	II	T2N1M0	Luminal B	Figures 4 and 5
40	IDC	III	T1N2M0	TNBC	Figures 4 and 5
41	IDC	III	T2N2M0	TNBC	Figures 4 and 5
42	IDC	II	T2N1M0	Luminal A	Figures 4 and 5
43	IDC	II	T2N1M0	Luminal B	Figures 4 and 5
44	IDC	II	T1N0M0	Luminal A	Figures 4 and 5
45	IDC	I	T1N0M0	Luminal B	Figures 4 and 5
46	IDC	II	T2N0M0	TNBC	Figures 4 and 5
47	IDC	II	T2N1M0	Luminal B	Figures 4 and 5
48	IDC	II	T2N0M0	Luminal B	Figures 4 and 5
49	IDC	II	T1N1M0	Luminal B	Figures 4 and 5
50	IDC	II	T2N1M0	Luminal B	Figure 4
51	IDC	II	T2N1M0	Luminal B	Figures 4 and 5
52	IDC	I	T1N0M0	Luminal A	Figures 4 and 5
53	DCIS	0	TisN0M0	TNBC	Figures 4 and 5
54	IDC	I	T1N0M0	Luminal A	Figure 4
55	DCIS	0	TisN0M0	Luminal A	Figures 4 and 5
56	IDC	I	T1N0M0	TNBC	Figures 4 and 5
57	IDC	I	T1N0M0	Luminal B	Figures 4 and 5
58	IDC	I	T1N0M0	Luminal A	Figures 4 and 5
59	IDC	I	T1N0M0	Luminal A	Figures 4 and 5
60	DCIS	0	TisN0M0	Luminal B	Figures 4 and 5
FL3474	IDC	II	T2N1M0	Luminal B	Figures 4 and 5
YQ8170	IDC	III	T4N1M0	HER2	Figures 4 and 5
BCa-10657645	IDC	II	T2N1M0	Luminal B	Figure 3
BCa-10710318	IDC	II	T2N1M0	HER2	Figure 3
BCa-10659367	IDC	III	T2N2M0	HER2	Figure 3
BCa-01684222	IDC	II	Left: T2N1M0<br>Right: T1N1M0	Luminal B	Figure 3

Table S2 Clinical characterization of the late stage breast cancer patients included in the study

Patient ID	Histology	Stage	Metastatic or local recurrence site	Subtype	Data used in figures
M1	IDC	Late stage, distant metastasis	Bone	Luminal A	Figure 4
M2	IDC	Late stage, distant metastasis	Lung	TNBC	Figure 4
M3	IDC	Late stage, distant metastasis	Bone, lung, lymph node	Luminal A	Figure 4
M4	IDC	Late stage, distant metastasis	Bone	Luminal B	Figure 4
M5	IDC	Late stage, distant metastasis	Liver	HER-2	Figure 4
M6	IDC	Late stage, distant metastasis	Lung, bone, brain, ovarium	HER-2	Figure 4
M7	IDC	Late stage, distant metastasis	Lung	HER-2	Figure 4
M8	IDC	Late stage, distant metastasis	Lung	HER-2	Figure 4
M9	IDC	Late stage, distant metastasis	Liver, bone, ovarium	Luminal B	Figure 4
M10	IDC	Late stage, distant metastasis	Brain	Luminal B	Figure 4
M11	IDC	Late stage, distant metastasis	Lung, bone	Luminal A	Figure 4
M12	IDC	Late stage, distant metastasis	Liver, chest wall	Luminal B	Figure 4
M13	IDC	Late stage, distant metastasis	Bone, lymph node	HER-2	Figure 4
M14	IDC	Late stage, distant metastasis	Liver, lung	Luminal B	Figure 4
M15	IDC	Late stage, distant metastasis	Contralateral breast	TNBC	Figure 4
M16	IDC	Late stage, distant metastasis	Lymph node, liver, lung, brain	HER-2	Figure 4
M17	IDC	Late stage, distant metastasis	Bone, ascites	Luminal A	Figure 4
M18	IDC	Late stage, distant metastasis	Brain	Luminal B	Figure 4
M19	IDC	Late stage, distant metastasis	Bone, lung	Luminal A	Figure 4
M20	IDC	Late stage, distant metastasis	Lung	Luminal B	Figure 4
M21	IDC	Late stage, distant metastasis	Lymph node, contralateral breast, liver, lung	Luminal B	Figure 4
M22	IDC	Late stage, distant metastasis	Bone, lung, pleura, lymph node, chest wall	TNBC	Figure 4
R1	IDC	Late stage, local recurrence	Chest wall	HER-2	Figure 4
R2	IDC	Late stage, local recurrence	Lymph node	TNBC	Figure 4
R3	IDC	Late stage, local recurrence	Chest wall, lymph node	HER-2	Figure 4
R4	IDC	Late stage, local recurrence	Lymph node	HER-2	Figure 4
R5	IDC	Late stage, local recurrence	Chest wall	Luminal B	Figure 4
R6	IDC	Late stage, local recurrence	Chest wall	Luminal B	Figure 4
R7	IDC	Late stage, local recurrence	Chest wall	Luminal B	Figure 4
BCa-09303473	IDC	Late stage, local recurrence	Bone, liver	HER2	Figure 3

Table S3 Primary and secondary antibodies used in the study

Antibodies	Product number	Manufacturer
Primary antibodies		
Antibodies for epithelial markers		
EpCAM	AF960	R&D, USA
E-cadherin	AF648	R&D, USA
CK8	sc-241376	Santa Cruz, USA
CK18	sc-31700	Santa Cruz, USA
CK19	sc-33119	Santa Cruz, USA
Antibodies for mesenchymal markers		
Vimentin	5741	Cell Signaling, USA
Fibronectin	ab32419	Abcam, USA
N-Cadherin	ARE6045	Antibody Revolution, USA
Conjugated antibodies for epithelial and mesenchymal markers		
CDH1	562526	BD Pharmingen, USA
EpCAM	FAB9601	R&D, USA
Vimentin	562338	BD Pharmingen, USA
Fibronectin	563100	BD Pharmingen, USA
CDH2	562119	BD Pharmingen, USA
Other antibody		
HER2	OAPB00881	Aviva Systems Biology, USA
Secondary antibodies		
Donkey anti-goat IgG-Alexa Fluor 488	ab150129	Abcam, USA
Donkey anti-rabbit IgG-Alexa Fluor 647	ab150075	Abcam, USA
Donkey anti-rabbit IgG-Alexa Fluor 488	ab150073	Abcam, USA
Goat anti-chicken IgY-Alexa Fluor 647	ab150171	Abcam, USA
Donkey anti-sheep IgG-Alexa Fluor 647	713-605-003	Jackson ImmunoResearch, USA

Table S4 Primers used in qRT-PCR

Gene name		Sequence(5′ → 3′)
CDH1	F	ATTTTTCCCTCGACACCCGAT
	R	TCCCAGGCGTAGACCAAGA
EPCAM	F	AATCGTCAATGCCAGTGTACTT
	R	TCTCATCGCAGTCAGGATCATAA
KRT8	F	CAGAAGTCCTACAAGGTGTCCA
	R	CTCTGGTTGACCGTAACTGCG
KRT18	F	TCGCAAATACTGTGGACAATGC
	R	GCAGTCGTGTGATATTGGTGT
KRT19	F	ACCAAGTTTGAGACGGAACAG
	R	CCCTCAGCGTACTGATTTCCT
FN1	F	CGGTGGCTGTCAGTCAAAG
	R	AAACCTCGGCTTCCTCCATAA
VIM	F	AGTCCACTGAGTACCGGAGAC
	R	CATTTCACGCATCTGGCGTTC
CDH2	F	TGCGGTACAGTGTAACTGGG
	R	GAAACCGGGCTATCTGCTCG
CD45	F	ATTACCTGGAATCCCCCTCAAA
	R	TTGTGAAATGACACATTGCAGC

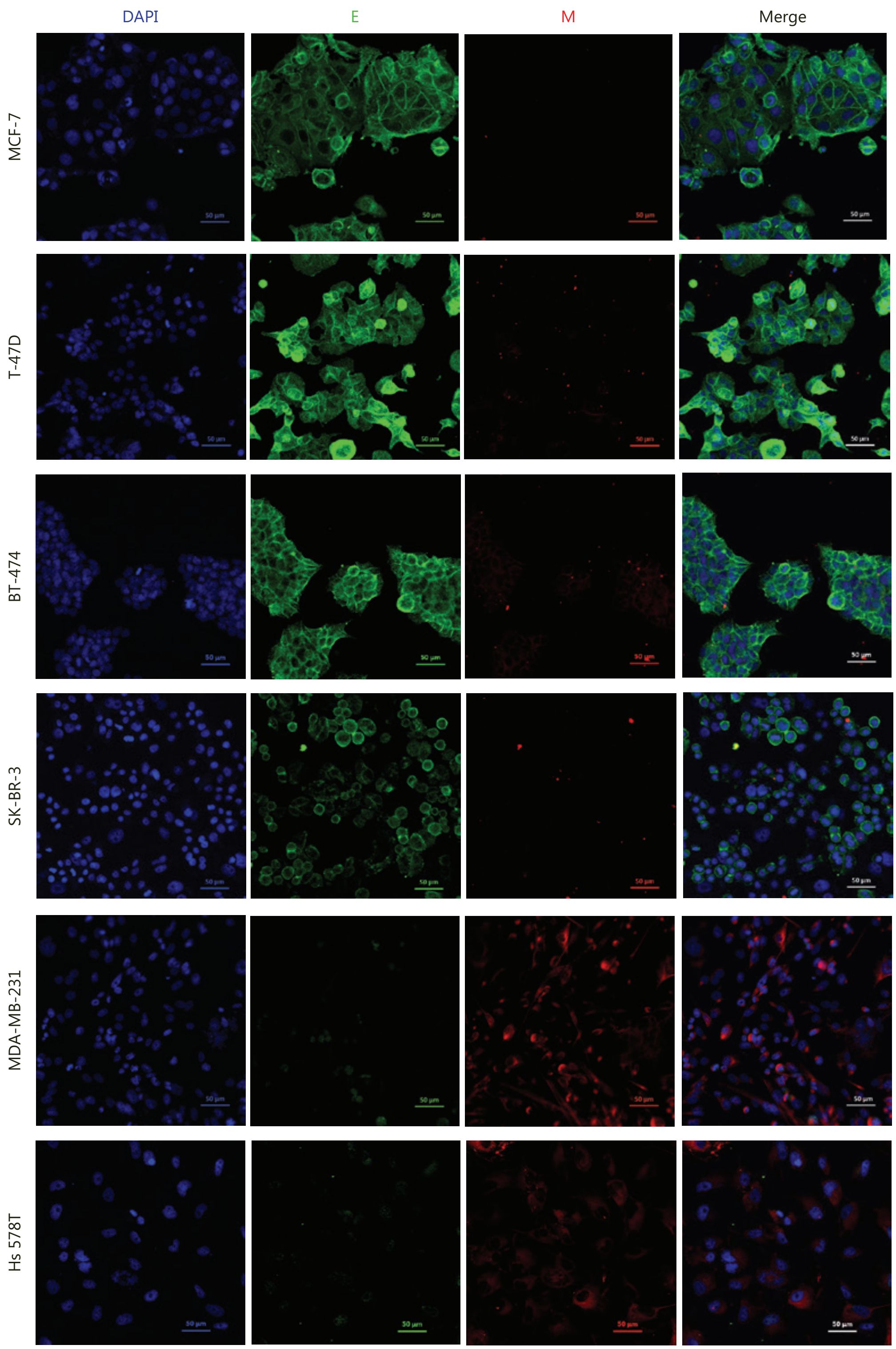

Figure S1 Testing of the antibodies for "E" (epithelial: EpCAM, E-cadherin, CK8, CK18, and CK19) and "M" (mesenchymal: vimentin, fibronectin, and N-cadherin) markers used in the negFACS-IF:E/M platform in various breast cell lines (MCF-7, T-47D, BT-474, SK-BR-3, MDA-MB-231, and Hs578T).

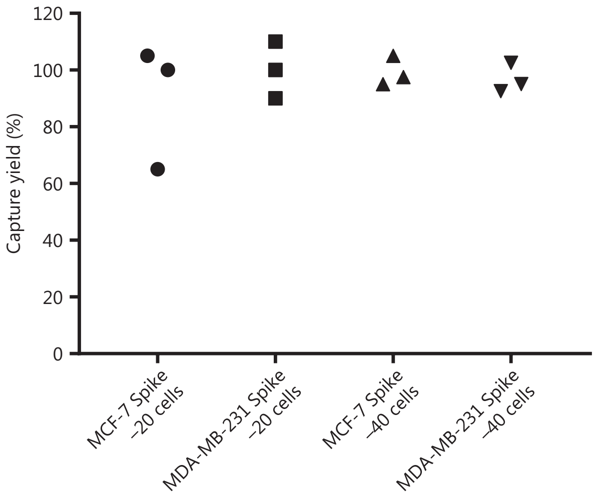

Figure S2 Performance recovery of the negFACS-IF:E/M platform. The capture yields (mean ± SD) for 20 cells and 40 cells of spiked MCF-7 cells (representative for "E" CTCs) were 90% ± 21.8% and 99% ± 5.2%, respectively, while those of spiked MDA-MB-231 cells (representative for "M" CTCs) were 100% ± 10% and 97% ± 5.2%, respectively. CTC: circulating tumor cells; SD: standard deviation; E CTCs: epithelial CTCs; M CTCs: mesenchymal CTCs.

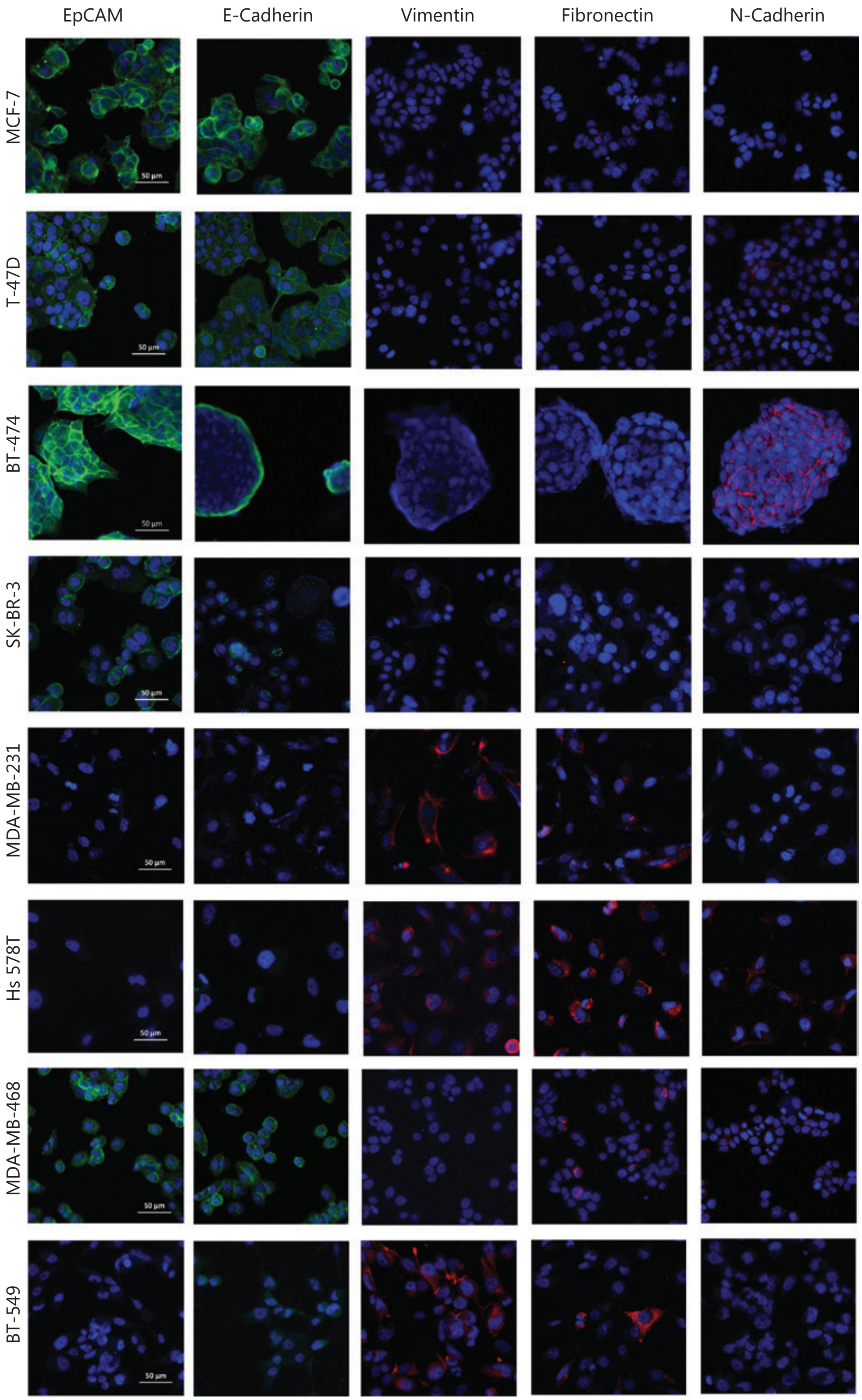

Figure S3 EMT status depicted by a panel of canonical epithelial (EpCAM, E-cadherin) and mesenchymal markers (vimentin, fibronectin, and N-cadherin) in various breast cancer cell lines (MCF-7, T-47D, BT-474, SK-BR-3, MDA-MB-231, Hs 578T, MDA-MB-468, and BT-549). The expression of different epithelial markers and mesenchymal markers varied considerably. EMT: epithelial-mesenchymal transition.

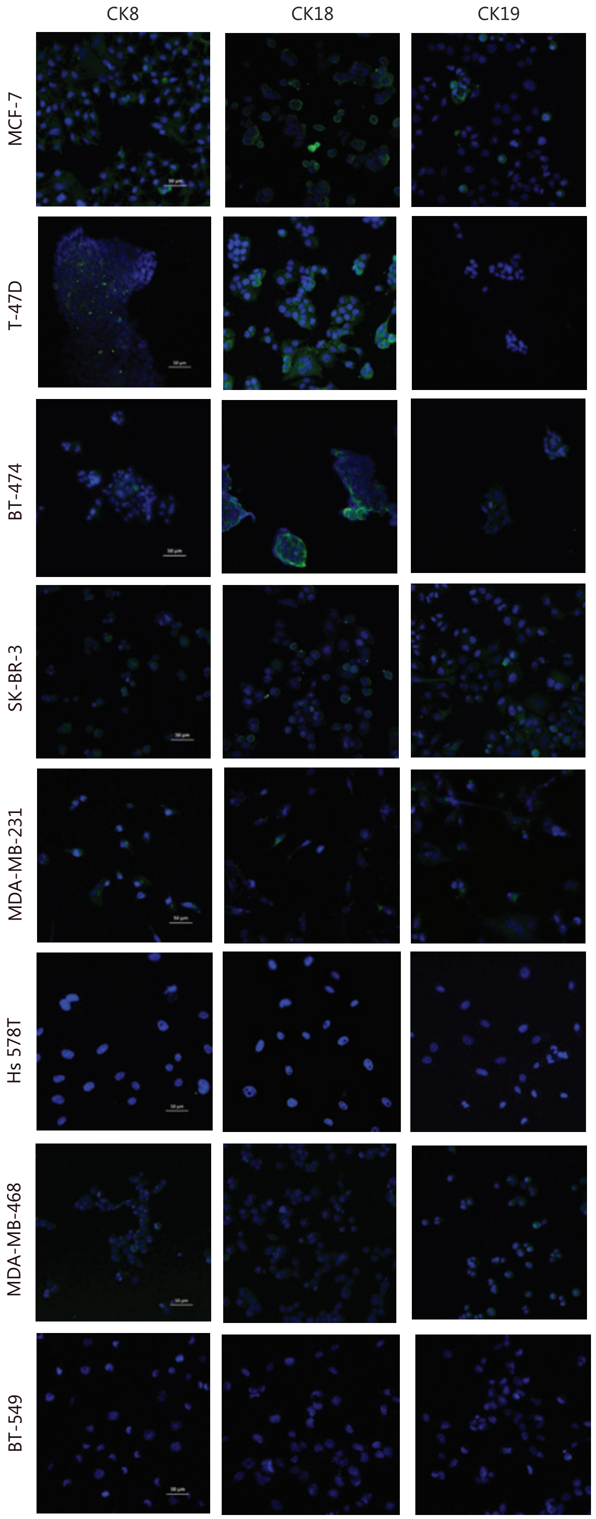

Figure S4 The expression of a panel of canonical epithelial (CK8, 18, and 19) markers in various breast cancer cell lines (MCF-7, T-47D, BT-474, SK-BR-3, MDA-MB-231, Hs 578T, MDA-MB-468, and BT-549). The expression of cytokeratins varied in different breast cancer cell lines.
